# Hydrogen Sulfide Alleviates Lipopolysaccharide-Induced Diaphragm Dysfunction in Rats by Reducing Apoptosis and Inflammation through ROS/MAPK and TLR4/NF-*κ*B Signaling Pathways

**DOI:** 10.1155/2018/9647809

**Published:** 2018-05-24

**Authors:** Guo-Yu Zhang, Dan Lu, Shao-Feng Duan, Ying-Ran Gao, Shi-Yu Liu, Ya Hong, Peng-Zhen Dong, Ya-Ge Chen, Tao Li, Da-Yong Wang, Xiang-Shu Cheng, Fei He, Jian-She Wei, Guang-Yu Li, Qing-Yong Zhang, Dong-Dong Wu, Xin-Ying Ji

**Affiliations:** ^1^The First Affiliated Hospital of Henan University, Kaifeng, Henan 475001, China; ^2^School of Basic Medical Sciences, Henan University College of Medicine, Kaifeng, Henan 475004, China; ^3^Henan International Joint Laboratory for Nuclear Protein Regulation, Henan University, Kaifeng, Henan 475004, China; ^4^College of Pharmacy, Henan University, Kaifeng, Henan 475004, China; ^5^Huaihe Hospital of Henan University, Kaifeng, Henan 475000, China; ^6^Brain Research Laboratory, College of Life Sciences, Henan University, Kaifeng, Henan 475004, China; ^7^Department of Microbiology and Immunology, School of Medicine, University of Texas Medical Branch, Galveston, TX 77555, USA; ^8^The Second Affiliated Hospital of Zhengzhou University, Zhengzhou, Henan 450014, China; ^9^Henan Provincial People's Hospital Affiliated to Henan University, Zhengzhou, Henan 450003, China

## Abstract

Diaphragm dysfunction is an important clinical problem worldwide. Hydrogen sulfide (H_2_S) is involved in many physiological and pathological processes in mammals. However, the effect and mechanism of H_2_S in diaphragm dysfunction have not been fully elucidated. In this study, we detected that the level of H_2_S was decreased in lipopolysaccharide- (LPS-) treated L6 cells. Treatment with H_2_S increased the proliferation and viability of LPS-treated L6 cells. We found that H_2_S decreased reactive oxygen species- (ROS-) induced apoptosis through the mitogen-activated protein kinase (MAPK) signaling pathway in LPS-treated L6 cells. Administration of H_2_S alleviated LPS-induced inflammation by mediating the toll-like receptor-4 (TLR-4)/nuclear factor-kappa B (NF-*κ*B) signaling pathway in L6 cells. Furthermore, H_2_S improved diaphragmatic function and structure through the reduction of inflammation and apoptosis in the diaphragm of septic rats. In conclusion, these findings indicate that H_2_S ameliorates LPS-induced diaphragm dysfunction in rats by reducing apoptosis and inflammation through ROS/MAPK and TLR4/NF-*κ*B signaling pathways. Novel slow-releasing H_2_S donors can be designed and applied for the treatment of diaphragm dysfunction.

## 1. Introduction

The diaphragm is a dome-shaped skeletal muscle in mammals that is mainly required for respiratory function [1, 2]. Pleural pressure is decreased by the contraction of the diaphragm, which makes the inspiration step of ventilation possible. Therefore, the efficiency of respiration is partly determined by the contractile performance of the diaphragm [3]. Diaphragm dysfunction is involved in a number of clinical conditions, including interstitial lung disease, chronic obstructive pulmonary disease, heart failure, spinal cord injury, critical illness and mechanical ventilation, and neuromuscular disease [4–9]. An increasing number of studies indicate that diaphragm dysfunction has been linked to impaired exercise tolerance, increased breathlessness, prolonged and difficult weaning from mechanical ventilation, and adverse health outcomes [10–12].

Hydrogen sulfide (H_2_S) has been considered the third gaseous signaling molecule, accompanying nitric oxide, and carbon monoxide [13, 14]. H_2_S can be endogenously produced from L-cysteine (L-Cys) and homocysteine in mammals mainly by two pyridoxal-5′-phosphate- (PLP-) dependent enzymes, namely cystathionine *γ*-lyase (CSE) and cystathionine *β*-synthase (CBS) [15–17]. 3-Mercaptopyruvate sulfurtransferase (3-MST), a PLP-independent enzyme, acts in combination with cysteine aminotransferase to produce H_2_S from L-Cys in the presence of *α*-ketoglutarate [15, 18]. There is increasing evidence that H_2_S plays important roles in a wide range of physiological and pathological conditions [13, 16, 18]. In the respiratory system, H_2_S has been shown to regulate many important functions such as pulmonary circulation, airway tone, fibrosis, cell proliferation or apoptosis, oxidative stress, and inflammation [19–21]. However, the effect and mechanism of H_2_S in diaphragm dysfunction have not been fully elucidated.

Sepsis is a life-threatening organ dysfunction caused by a dysregulated host response to infection [22, 23]. A number of studies have indicated that sepsis could induce diaphragm dysfunction, which can be attributed to the localized elaboration of cytokines within the diaphragm [24–26]. Lipopolysaccharide (LPS) is one of the cell wall components of most Gram-negative bacteria and acts as a potent initiator of inflammation [27, 28]. Recently, LPS has been widely adopted to induce septic diaphragm dysfunction in many different animal models [24, 27, 29]. In the present study, we detected the protein expressions of H_2_S-generating enzymes and the level of endogenous H_2_S in LPS-treated skeletal muscle cells, and further determined the effect of exogenous H_2_S on diaphragm dysfunction and clarified the associated molecular mechanism.

## 2. Materials and Methods

### 2.1. Cell Culture

Rat skeletal muscle cell line L6 was obtained from the Institute of Biochemistry and Cell Biology, Chinese Academy of Sciences (IBCB, CAS, Shanghai, China) and maintained in Dulbecco's modified Eagle's medium (DMEM) supplemented with 10% fetal calf serum, 100 U/ml penicillin, and 100 *μ*g/ml streptomycin. Cells were cultured in a humidified incubator with 5% CO_2_ and 95% air at 37°C. Confluent L6 cells were transferred to serum-free DMEM medium for overnight starvation before each experiment. The cells were divided into three groups: control group, LPS group, and LPS + H_2_S group. The control group was treated with phosphate-buffered saline (PBS) and the LPS group was treated with 250 *μ*g/ml LPS (dissolved in PBS). The LPS + H_2_S group was treated with 250 *μ*g/ml LPS and 100 *μ*M NaHS (an H_2_S donor, dissolved in PBS). After 24 h of treatment, the cells were then used for subsequent experiments.

### 2.2. Measurement of H_2_S Levels

The concentrations of H_2_S in L6 cells and culture supernatant were measured using an enzyme-linked immunosorbent assay (ELISA) kit (LanpaiBio, Shanghai, China) as previously described [30].

### 2.3. Cell Growth Assay

The 5-ethynyl-2′-deoxyuridine (EdU) incorporation assay was performed using the Cell-Light EdU Apollo 567 *In Vitro* Imaging Kit (RiboBio, Guangzhou, Guangdong, China) according to the manufacturer's instructions. Cell proliferation rate = (EdU-positive cells)/(total number of cells) × 100%. The cell viability was detected using the CellTiter 96 AQ_ueous_ One Solution Cell Proliferation Assay kit (MTS; Promega, Madison, WI, USA) according to the manufacturer's protocols. The cell viability was expressed as a percentage of the untreated control.

### 2.4. Cellular Apoptosis Analysis

Cellular apoptosis was analyzed by the in situ terminal deoxynucleotidyl transferase-mediated dUTP nick end labeling (TUNEL) assay using a cell death detection kit (Beyotime Institute of Biotechnology, Shanghai, China) following the manufacturer's instructions. Briefly, cells were fixed with 4% paraformaldehyde, permeabilized with 0.1% Triton X-100, incubated with 50 *μ*l TUNEL reaction mixture for 60 min at 37°C in darkness and then rinsed with PBS three times. After counterstaining with 5 mg/ml DAPI for 5 min at room temperature, cells were photographed with a fluorescent microscope (Eclipse Ti, Nikon, Melville, NY, USA) from six random fields. The apoptotic index = (positively stained apoptotic cells)/(total number of cells) × 100%.

### 2.5. Detection of Intracellular Reactive Oxygen Species (ROS)

Intracellular ROS generation was detected by using a 2′,7′-dichlorofluorescin diacetate- (DCF-DA-) Cellular Reactive Oxygen Species Detection Assay Kit (Beyotime Institute of Biotechnology, Shanghai, China). Cells were incubated with 10 *μ*M DCF-DA for 30 min in darkness at 37°C and washed three times with PBS. The fluorescence was observed under a fluorescent microscope (Eclipse Ti, Nikon, Melville, NY, USA) from six random fields and measured by Image J software (National Institutes of Health, Bethesda, MD, USA).

### 2.6. Antioxidant Activity Determination

The activities of superoxide dismutase (SOD), glutathione peroxidase (GSH-Px), and catalase (CAT) in L6 cells were measured using commercial kits (Beyotime Institute of Biotechnology, Shanghai, China) according to the manufacturer's instructions.

### 2.7. Biochemical Analysis

The contents of IL-6, IL-10, and IL-18 in L6 cells were determined using commercial ELISA kits (Elabscience, Wuhan, Hubei, China) according to the manufacturer's protocols.

### 2.8. Western Blotting

Total protein was extracted from L6 cells. Western blotting was performed to detect the target proteins. The primary antibodies, including anti-CSE, anti-CBS, anti-3-MST, anti-B-cell lymphoma-2 (Bcl-2), anti-B-cell lymphoma-extra large (Bcl-xl), anti-Bcl-2-associated X protein (Bax), anti-Bcl-xl/Bcl-2-associated death promoter (Bad), anti-cleaved caspase-3, anti-cleaved caspase-8, anti-cleaved caspase-9, anti-cleaved polyadenosine diphosphate-ribose polymerase (PARP), and anti-glyceraldehyde-3-phosphate dehydrogenase (GAPDH) antibodies were purchased from Proteintech (Chicago, IL, USA). Anti-extracellular signal-regulated protein kinase 1/2 (ERK1/2), anti-phospho (p)-ERK1/2 (Thr202/Tyr204), anti-p38, anti-p-p38 (Thr180/Tyr182), anti-c-Jun N-terminal kinase (JNK), anti-p-JNK (Thr183/Tyr185), anti-toll-like receptor-4 (TLR-4), anti-transforming growth factor-activated kinase-1 (TAK1), anti-p-TAK1 (Ser412), anti-inhibitor of nuclear factor kappa-B kinase (IKK) subunit alpha (IKK*α*), anti-IKK subunit *β* (IKK*β*), anti-p-IKK*α*/*β* (Ser176/180), anti-nuclear factor of kappa light polypeptide gene enhancer in B-cell inhibitor, alpha (I*κ*B*α*), anti-p-I*κ*B*α* (Ser32), anti-p50, anti-p65, anti-p-p65 (Ser536), and the horseradish peroxidase-conjugated secondary antibody were purchased from Cell Signaling Technology (CST, Danvers, MA, USA). Results were normalized to the level of GAPDH. The reaction was visualized using an enhanced chemiluminescence system (Thermo Fisher Scientific, Rockford, IL, USA). The bands were semiquantified with Image J software.

### 2.9. Animal Study

Animal experiments were approved by the Committee of Medical Ethics and Welfare for Experimental Animals of Henan University School of Medicine in compliance with the Experimental Animal Regulations formulated by the National Science and Technology Commission, China (HUSOM-2017-198). All animal experiments were conducted in accordance with the committee's approved guidelines. Eighteen male Wistar rats (6–8 weeks old), initially weighing 180–220 g, were purchased from Beijing Vital River Laboratory Animal Technology Co., Ltd. (Beijing, China). Rats were housed in individual cages in a temperature- and humidity-controlled room with a 12-hour light/dark cycle. All rats were fed standard pellet food and distilled water ad libitum and allowed to acclimatize to new surroundings for one week prior to the study. Animal studies were performed as previously described with minor modifications [24]. Briefly, rats received an intraperitoneal (i.p.) injection of 5 mg/kg LPS which was dissolved in normal saline (NS) to induce diaphragm dysfunction. A normal control group was also allocated. Subsequently, the rats from the control and LPS groups received an i.p. injection of NS and the rats from the LPS + H_2_S group received an i.p. injection of NaHS (100 *μ*mol/kg, dissolved in NS).

### 2.10. Diaphragm Contractile Measurements

Twenty-four hours after the administration of NaHS or NS, all rats were killed and the body weight change was measured. The right hemidiaphragm was quickly removed and immediately immersed in Krebs solution that was continuously aerated with 95% O_2_/5% CO_2_ and contained the following (mmol/l): 135 NaCl, 5 KCl, 2.5 CaCl_2_, 1 MgSO_4_, 1 NaH_2_PO_4_, 15 NaHCO_3_, and 1 glucose, with a pH of 7.4. A diaphragm muscle strip (5 mm wide) was obtained from the midcostal region of the right diaphragm by careful dissection parallel to the long axis of the fibers. The muscle strip was suspended vertically in a 37°C tissue bath containing Krebs solution. The end of the muscle was tied to a rigid support. The muscle tendon was connected to an isometric force transducer mounted on a micrometer.

Two silver stimulating electrodes were placed parallel to the muscle strip. After equilibration for 15 min, isometric contractions were recorded at the optimal muscle length (L0) at which the peak twitch force was observed. Stimuli were applied using a rectangular 0.2 ms duration pulse and a train duration of 250 ms. To ensure supramaximal stimulation, the strips were stimulated at 20% above voltage to obtain maximal forces. The signal was amplified and recorded using a data acquisition system (MedLab, Nanjing, Jiangsu, China). Once maximal stimulus intensity and the L0 for force production were determined, peak twitch tension was determined at L0 from a series of contractions induced by single-pulse stimuli and maximal rate of contraction (+*dT*/*dt*_max_) and maximal rate of relaxation (−*dT*/*dt*_max_) were measured. Maximal tetanic tension was produced by a supramaximal 250 ms stimulus train at 100 Hz. To measure the force-frequency response, each strip was stimulated with a 250 ms train at 10, 20, 40, 60, and 100 Hz, with at least a 2 min interval between each stimulus train. The fatigue index of the diaphragm was assessed by a series of 150 tetanic contractions (400 ms contraction times at 10, 20, 40, 60, and 100 Hz once every second). The fatigue index was calculated from the ratio of the force produced during the 150th contraction relative to the 1st contraction [31]. After the measurements were taken, the muscle strip was blotted dry and weighed. The forces were normalized to muscle cross-sectional area (CSA) and expressed as specific force (N/cm^2^). Tissue CSA was estimated using measurements of muscle length (cm), mass (g), and density (1.056 g/cm^3^). The left costal hemidiaphragm was used for biochemical and molecular experiments, or embedded in FSC 22 frozen section compound (Leica, Buffalo Grove, IL, USA) for histological staining.

### 2.11. Quantitative Real-Time Polymerase Chain Reaction (qRT-PCR)

Total RNA was isolated from diaphragmatic muscle tissues using a TRIzol reagent, treated with DNase I, and purified using an RNA clean-up kit (Cwbiotech, Beijing, China). Total RNA (1 *μ*g) was applied for cDNA synthesis using a cDNA reverse transcription kit (Cwbiotech, Beijing, China). Primers were designed according to the primer design principles with Primer Premier 5.0 (Premier Biosoft, Palo Alto, CA, USA). The sequences of primers were listed in Table 1. The reactions were performed in a total volume of 20 *μ*l using the following thermal cycling parameters: 95°C for 10 min, 40 cycles of 95°C for 15 s, 60°C for 60 s, and 72°C for 1 min. The results were normalized to the level of GAPDH.

### 2.12. Histological Analysis

A necropsy examination was performed immediately after the rats were sacrificed. Diaphragmatic muscle tissues were fixed in 10% neutral buffered formalin, embedded in paraffin, sectioned at 5 *μ*m thickness, and processed according to the hematoxylin and eosin (HE) staining protocols. Diaphragmatic muscle tissues were then stained with anti-myosin heavy chain (MHC) Type I, anti-MHC Type IIa, anti-Ki67, and anti-cleaved caspase 3 antibodies (CST, Danvers, MA, USA), followed by incubation with a secondary antibody. Diaphragmatic muscle tissues were observed using a Zeiss Axioskop 2 plus microscope (Carl Zeiss, Thornwood, NY, USA). The cross-sectional area (CSA) of a diaphragm muscle fiber was determined according to a previous study [32]. The proliferation index (PI) was determined by the number of Ki67 positive cells among the total number of cells [33]. Furthermore, apoptosis rate was estimated as cleaved caspase 3 positive cells/total cells [34].

### 2.13. Statistical Analysis

All results were presented as the mean ± standard error of the mean (SEM). Statistical differences were analyzed by one-way analysis of variance (ANOVA) using SPSS 17.0 software, followed by an LSD post hoc test. A *P* value of less than 0.05 was considered to be statistically significant.

## 3. Results

### 3.1. The Level of H_2_S Is Decreased in LPS-Treated L6 Cells and Administration of H_2_S Increases the Growth of LPS-Treated L6 Cells

**Figure 1:**
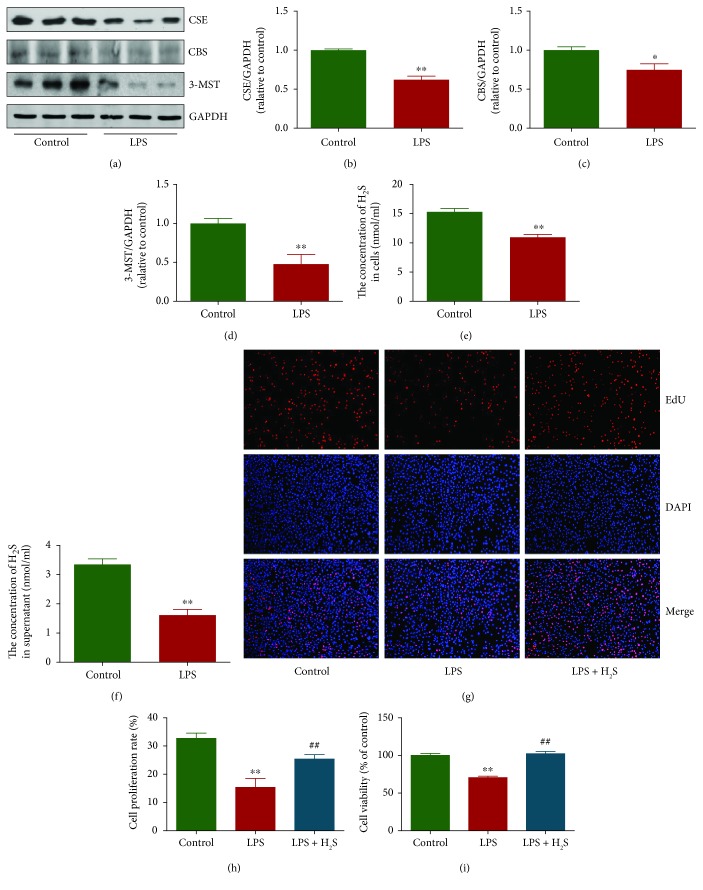
The levels of endogenous H_2_S in L6 cells and LPS-treated L6 cells were detected, and the effects of exogenous H_2_S on the growth of LPS-treated L6 cells were examined. (a) The protein expression of CSE, CBS, and 3-MST were examined by Western blot. GAPDH was used as the loading control. (b–d) Bar graphs showed the quantification of CSE, CBS, and 3-MST. The densitometry analysis of each factor was performed, normalized to the corresponding GAPDH level. (e) The levels of H_2_S in L6 cells and LPS-treated L6 cells. (f) The levels of H_2_S in the culture supernatant. (g) DNA replication activities were examined by EdU assay; original magnification, ×200. (h) The proliferation rate of each group was analyzed. (i) The percentages of viable cells were determined using the MTT assay, and the cell viability of L6 cells was normalized as 100% and considered to be the control group. Data are presented as mean ± SEM of three independent experiments; ^∗^*P* < 0.05 and ^∗∗^*P* < 0.01 compared with the control group; ^##^*P* < 0.01 compared with the LPS group.

As shown in Figures 1(a)–1(d), the protein levels of CSE, CBS, and 3-MST in LPS-treated L6 cells were lower than those in untreated L6 cells. In addition, the levels of H_2_S in LPS-treated L6 cells, as well as in the supernatant, were lower than those in untreated L6 cells and the supernatant (Figures 1(e) and 1(f)). These results suggest that H_2_S may play an important role in the growth of L6 cells. To test this hypothesis, we determined the effect of exogenous H_2_S on the growth of LPS-treated L6 cells. The proliferation and viability of L6 cells were inhibited by the treatment of LPS, while 100 *μ*M NaHS enhanced the proliferation and viability of LPS-treated L6 cells (Figures 1(g)–1(i)).

### 3.2. H_2_S Reduces Apoptosis in LPS-Treated L6 Cells

**Figure 2:**
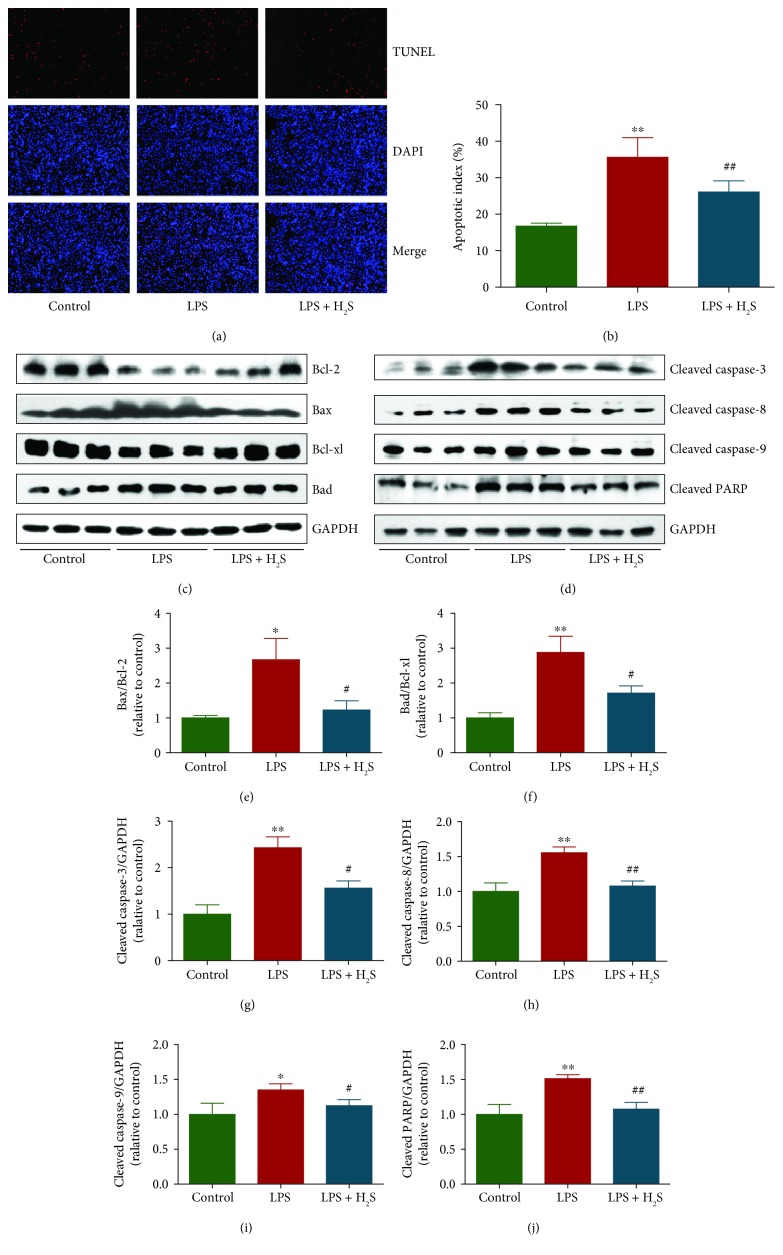
Effects of H_2_S on the apoptosis of LPS-treated L6 cells. (a) The apoptotic levels were measured by TUNEL staining; original magnification, ×100. (b) The percentages of TUNEL-positive cells were calculated by the formula: the apoptotic index = (positively stained apoptotic cells)/(total number of cells) × 100%. (c–d) Western blotting analysis for the expression levels of Bcl-2, Bax, Bcl-xl, and Bad, cleaved caspase-3, -8, and -9 and cleaved PARP in each group. GAPDH was used as the loading control. (e–j) The densitometry analysis of each factor was performed in each group, normalized to the corresponding GAPDH level. Data are presented as mean ± SEM of three independent experiments; ^∗^*P* < 0.05 and ^∗∗^*P* < 0.01 compared with the control group; ^#^*P* < 0.05 and ^##^*P* < 0.01 compared with the LPS group.

As shown in Figures 2(a) and 2(b), the apoptotic index increased in the LPS group compared with the control group. Administration of H_2_S decreased the apoptotic index in the LPS group. The ratios of Bax/Bcl-2 and Bad/Bcl-xl have been widely considered important factors in the regulation of apoptosis. In mammalian cells, increased Bad/Bcl-xl and Bax/Bcl-2 ratios are common phenomena in mitochondrial apoptosis [35, 36]. As shown in Figures 2(c), 2(e), and 2(f), the Bax/Bcl-2 and Bad/Bcl-xl ratios increased in the LPS group compared with the control group and decreased in the LPS + H_2_S group compared with the LPS group. Furthermore, the protein expression levels of cleaved caspase-3, -8, and -9 and cleaved PARP showed similar trends (Figures 2(d), 2(g)–2(j)). In sum, these results indicate that the apoptotic level in LPS-treated L6 cells is increased and administration of H_2_S significantly reduces the apoptotic level.

### 3.3. H_2_S Mediates the ROS/Mitogen-Activated Protein Kinase (MAPK) Signaling Pathway in L6 Cells

**Figure 3:**
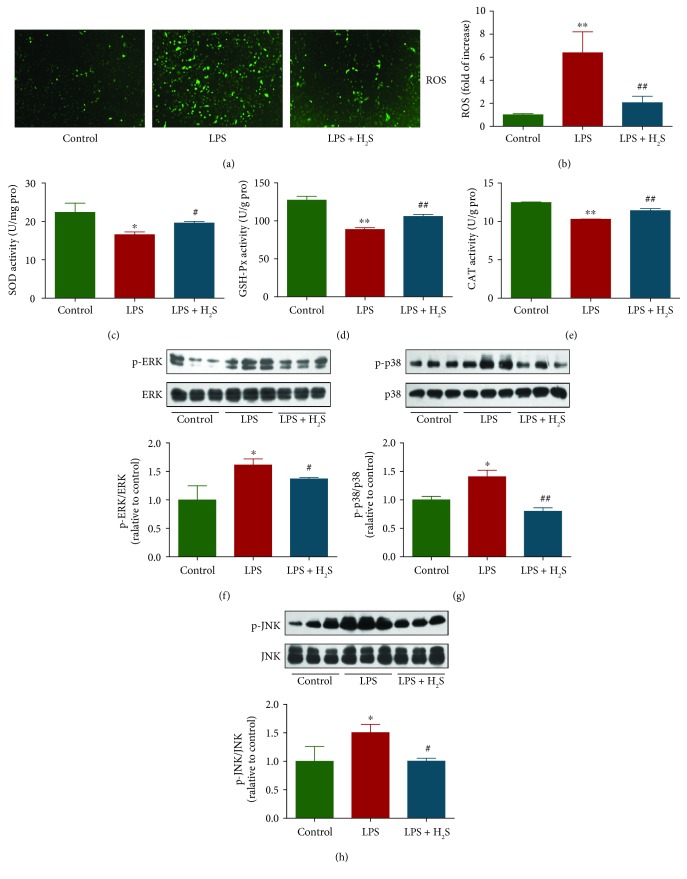
Effects of H_2_S on the ROS/MAPK signaling pathway in LPS-treated L6 cells. (a) The intracellular ROS production was detected using the fluorescent probe DCF-DA (shown in green; original magnification, ×100). (b) The intracellular ROS production was measured. (c–e) The activities of SOD, GSH-Px, and CAT were measured. (f–h) The protein expressions of ERK1/2, p-ERK1/2, p38, p-p38, JNK, and p-JNK were analyzed by Western blotting. Data are presented as mean ± SEM of three independent experiments; ^∗^*P* < 0.05 and ^∗∗^*P* < 0.01 compared with the control group; ^#^*P* < 0.05 and ^##^*P* < 0.01 compared with the LPS group.

ROS are formed upon partial reduction of oxygen, including hydrogen peroxide, superoxide anion, singlet oxygen, and hydroxyl radical [37]. Enzymatic ROS-scavenging mechanisms include a number of antioxidant enzymes such as SOD, GSH-Px, and CAT [37, 38]. Compared to the control group, the levels of ROS were increased, and the activities of SOD, GSH-Px, and CAT were decreased in the LPS group, which were all reversed by treatment with H_2_S (Figures 3(a)–3(e)). These results suggest that H_2_S is able to abate LPS-induced oxidative stress in L6 cells. ROS are important and common messengers produced in various environmental stresses and are known to activate the MAPK pathway [37, 39, 40]. The MAPK family is composed of three major components: ERK, JNK, and p38 protein kinases [41, 42]. As shown in Figures 3(f)–3(h), LPS triggered phosphorylations of p38, JNK, and ERK with distinct patterns. However, treatment with H_2_S decreased phosphorylations of these protein kinases. The results show that H_2_S mediates the ROS/MAPK pathway in L6 cells.

### 3.4. H_2_S Alleviates LPS-Induced Inflammation by Mediating the TLR4/Nuclear Factor-Kappa B (NF-*κ*B) Signaling Pathway in L6 Cells

**Figure 4:**
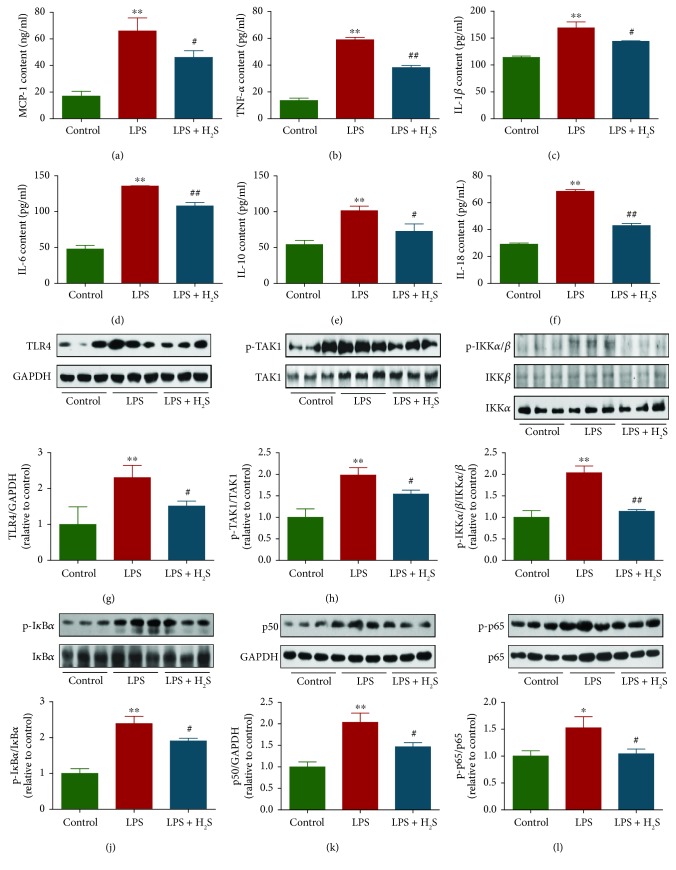
Effects of H_2_S on the cytokine levels and the TLR4/NF-*κ*B signaling pathway in diaphragmatic muscle tissues of septic rats. (a–f) The expression levels of MCP-1, TNF-*α*, IL-1*β*, IL-6, IL-10, and IL-18 were measured. (g–l) The protein expressions of TLR4, TAK1, p-TAK1, IKK*α*, IKK*β*, p-IKK*α*/*β*, I*κ*B*α*, p-I*κ*B*α*, p50, p65, p-p65, and GAPDH were analyzed by Western blotting. Data are presented as mean ± SEM of three independent experiments; ^∗^*P* < 0.05 and ^∗∗^*P* < 0.01 compared with the control group; ^#^*P* < 0.05 and ^##^*P* < 0.01 compared with the LPS group.

Many studies have shown that treatment with LPS could induce increased expressions of inflammatory cytokines in L6 cells, such as MCP-1, TNF-*α*, IL-6, and IL-10 [43–45]. Whether H_2_S could reduce the levels of inflammation in LPS-treated L6 cells remains unknown. In this study, the inflammatory cytokine levels in L6 cells were determined using ELISA techniques. Compared with the control group, expression levels of MCP-1, TNF-*α*, IL-1*β*, IL-6, IL-10, and IL-18 were increased in the LPS group. Treatment with H_2_S decreased the levels of these inflammatory cytokines (Figures 4(a)–4(f)), suggesting that H_2_S could alleviate inflammation in LPS-treated L6 cells. TLR4, a type I transmembrane receptor, plays an important role in the innate immune system. It is widely accepted that LPS can activate the TLR4/NF-*κ*B signaling pathway [46, 47]. In the present study, we found that LPS increased the expression of TLR4, which was reduced by H_2_S treatment (Figure 4(g)). TAK1, IKK, and I*κ*B are key upstream modulators for NF-*κ*B activation [48]. As shown in Figures 4(h)–4(j), LPS treatment increased levels of phosphorylated TAK1, IKK*α*/*β*, and I*κ*B*α*. However, these effects were reversed by treatment with H_2_S. The most abundant form of NF-*κ*B is the heterodimer composed of p50 and p65 [49]. Compared with the control group, LPS treatment increased protein expressions of p50 and p-p65 and the p-p65/p65 ratio. Administration of H_2_S decreased expression levels of p50 and p-p65, as well as the ratio of p-p65/p65 (Figures 4(k) and 4(l)). These results together indicate that H_2_S is capable of reducing LPS-induced inflammation through inhibition of the TLR4/NF-*κ*B signaling pathway.

### 3.5. H_2_S Improves Diaphragmatic Function and Structure of Septic Rats

**Figure 5:**
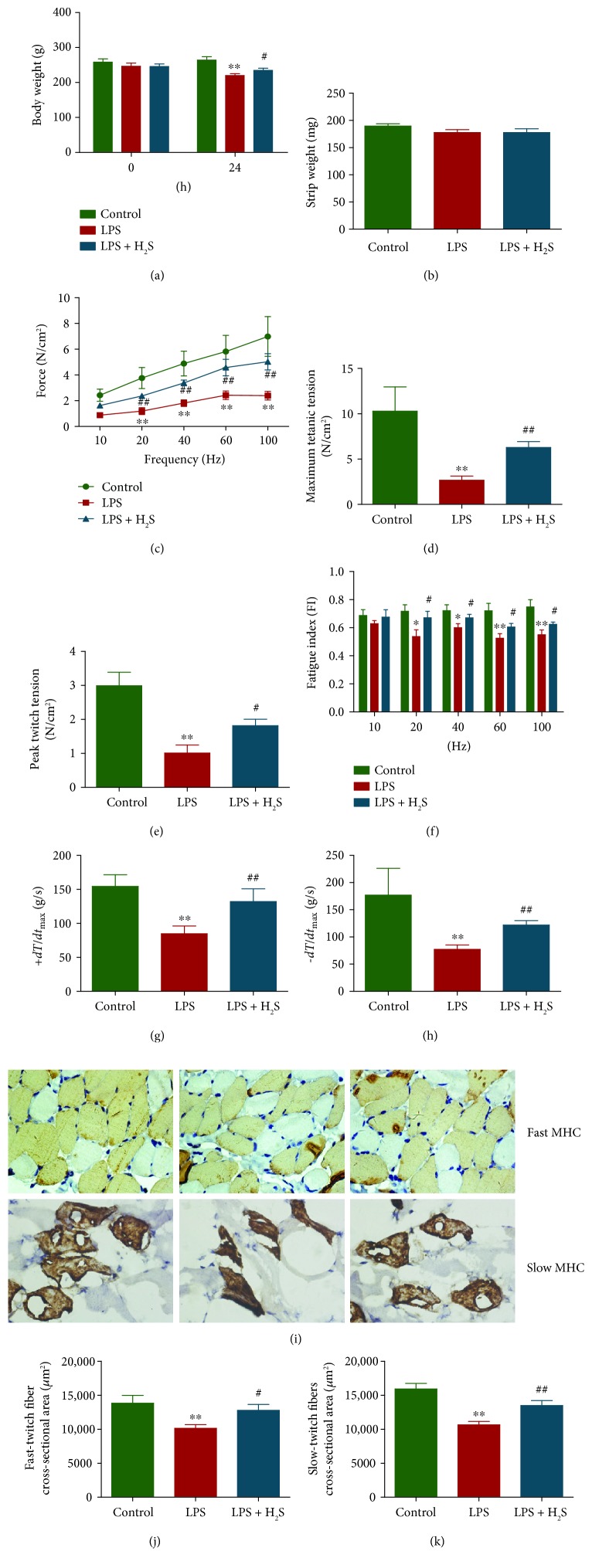
Effects of H_2_S on the diaphragmatic function and structure of septic rats. (a, b) The body weight and strip weight of rats were measured. (c) The contractile force was measured. (d, e) The maximum tetanic tension and peak twitch tension were detected. (f) The fatigue index was measured. (g, h) The maximal rate of contraction (+*dT*/*dt*_max_) and the maximal rate of relaxation (−*dT*/*dt*_max_) were detected. (i) Representative photographs of diaphragm muscle cross-sectional area in diaphragm skeletal muscle myofibers of rats; original magnification, ×400. (j, k) Fast-twitch fibers and slow-twitch fibers were calculated. Values were presented as mean ± SEM (*n* = 6). ^∗^*P* < 0.05 and ^∗∗^*P* < 0.01 compared with the control group; ^#^*P* < 0.05 and ^##^*P* < 0.01 compared with the LPS group.

There was no significant difference in body weight at the beginning of the animal study. After 24 h, LPS treatment reduced the body weights of the rats, while H_2_S increased the body weights of septic rats (Figure 5(a)). However, there was no significant difference in strip weight among each group (Figure 5(b)). LPS elicited a large reduction in the force-generating capacity of the diaphragm, producing dramatic reductions in muscle-specific force generation of the diaphragm. Administration of H_2_S attenuated the effects of LPS on diaphragm strength, with force generation at the stimulation frequencies from 20 to 100 Hz (Figure 5(c)). As shown in Figures 5(d) and 5(e), LPS decreased both maximum tetanic tension and peak twitch tension, whereas the effects were reversed by treatment with H_2_S. In addition, LPS reduced the fatigue index, which was enhanced by H_2_S treatment (Figure 5(f)). Furthermore, LPS decreased the maximal rates of contraction and relaxation (±*dT*/*dt*_max_), while H_2_S increased the maximal rates of contraction/relaxation (Figures 5(g) and 5(h)). As shown in Figures 5(i)–5(k), the CSA of both MHC_fast_ and MHC_slow_ was decreased in the LPS group. However, administration of H_2_S prevented the loss of the CSA of MHC_fast_ and MHC_slow_ compared with the LPS group, indicating that H_2_S can reduce LPS-induced diaphragm injury. Collectively, these data demonstrate that H_2_S improves diaphragmatic function and structure of septic rats.

### 3.6. H_2_S Decreases Inflammation and Apoptosis in the Diaphragm of Septic Rats

**Figure 6:**
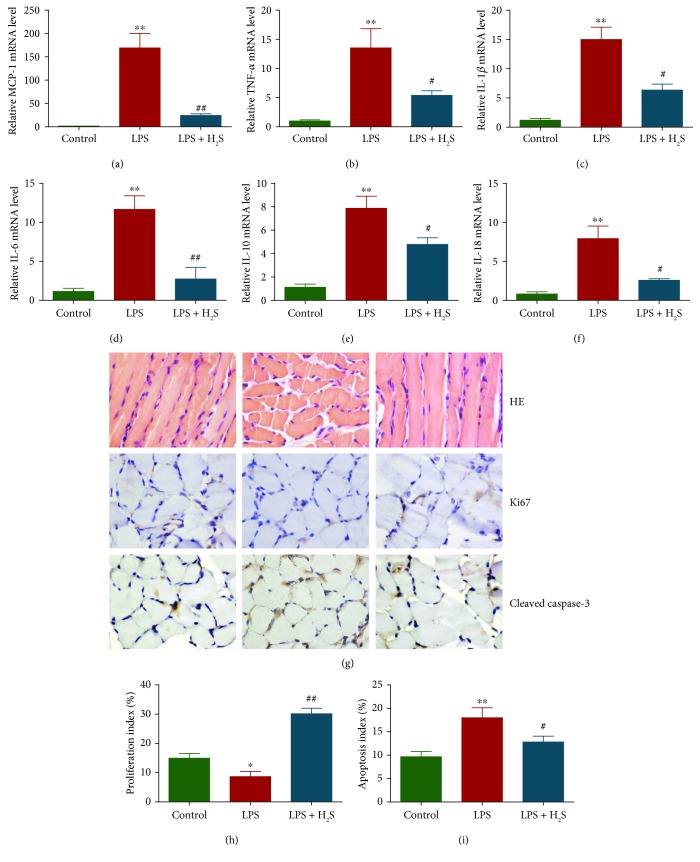
Effects of H_2_S on the inflammation, proliferation, and apoptosis in the diaphragm of septic rats. (a–f) The expressions of MCP-1, TNF-*α*, IL-1*β*, IL-6, IL-10, and IL-18 were detected by qRT-PCR. Data are presented as mean ± SEM of three independent experiments. (g) Representative photographs of HE, Ki67, and cleaved caspase-3 staining in the diaphragm of rats; original magnification, ×400. (h, i) The proliferation index and apoptosis index were calculated. Values were presented as mean ± SEM (*n* = 6). ^∗^*P* < 0.05 and ^∗∗^*P* < 0.01 compared with the control group; ^#^*P* < 0.05 and ^##^*P* < 0.01 compared with the LPS group.

The expressions of cytokine genes were detected by qRT-PCR. As shown in Figures 6(a)–6(f), gene expressions of MCP-1, TNF-*α*, IL-1*β*, IL-6, IL-10, and IL-18 were increased in the LPS group. Treatment with H_2_S decreased gene expressions of these inflammatory cytokines. IHC with the Ki67 antibody confirmed that the *in vivo* proliferation of diaphragmatic muscle cells was inhibited in the LPS group and promoted in the LPS + H_2_S group (Figures 6(g) and 6(h)). However, the protein expression of cleaved caspase-3 exhibited an opposite trend (Figures 6(g) and 6(i)). These results together indicate that H_2_S is able to decrease inflammation and apoptosis in the diaphragm of septic rats.

## 4. Discussion

H_2_S has been considered the third gaseous signaling molecule and plays important roles in a wide range of physiological and pathological conditions [13, 16, 18]. However, the effect and mechanism of H_2_S in diaphragm dysfunction have not been fully elucidated. Rat skeletal muscle cell line L6 has been widely used to study the regeneration and function of skeletal muscle [50–52]. LPS, a major constituent of the cell wall of Gram-negative bacteria, is a potent agent widely used to model bacterial challenges of mammalian cells [27, 28]. In the present study, L6 cells were used to evaluate the effects of H_2_S on the LPS-induced diaphragmatic injury *in vitro*. The results demonstrated that the protein levels of H_2_S-generating enzymes and the levels of H_2_S in LPS-treated L6 cells and supernatant were lower than those in L6 cells and the supernatant, suggesting that H_2_S may be involved in the growth of L6 cells. To test this hypothesis, we examined the effect of exogenous H_2_S on the growth of LPS-treated L6 cells. The results showed that the proliferation and viability of L6 cells were inhibited in the LPS group, whereas administration of H_2_S enhanced the proliferation and viability of LPS-treated L6 cells, indicating that H_2_S is involved in the growth of L6 cells.

Apoptosis is a highly regulated mechanism of cell death which plays a critical role in the normal development and maintenance of tissue homeostasis in multicellular organisms [53]. There are two main apoptotic signaling pathways: an extrinsic pathway initiated by death receptors and an intrinsic pathway that occurs through the mitochondria [54]. Apoptosis is regulated by Bcl-2 family proteins, including proapoptotic proteins such as Bax and Bad, and antiapoptotic proteins such as Bcl-2 and Bcl-xl [55]. Caspases can be activated by apoptotic stimuli and cleaved caspase-3 can inactivate PARP, thus leading to the occurrence of apoptotic cascade [56]. Recent studies have shown that LPS could induce apoptosis in different types of cells [28, 57, 58]. Similarly, our results indicated that LPS increased the apoptotic index, Bax/Bcl-2 and Bad/Bcl-xl ratios, as well as protein expression levels of cleaved caspase-3, -8, and -9 and cleaved PARP in L6 cells. Treatment with H_2_S decreased the apoptotic level in the LPS group. In sum, these results demonstrate that the apoptotic level in LPS-treated L6 cells is increased and administration of H_2_S can reduce the apoptotic level.

High levels of ROS could lead to an imbalance of the cellular redox state and oxidative stress, as well as induce cell apoptosis or necrosis in a number of physiological and pathological conditions [59, 60]. Our results demonstrated that LPS increased the levels of ROS and decreased the activities of SOD, GSH-Px, and CAT, which were similar to a previous study [61]. All these changes were reversed by treatment with H_2_S. It has been shown that ROS could activate MAPKs, and apoptotic cell death induced by ROS is mediated by the MAPK pathway [62]. The MAPK family is mainly composed of three components, including ERK, JNK, and p38 protein kinases [41, 42]. Recently, it has been demonstrated that LPS is able to decrease the protein synthetic rate in C2C12 mouse myoblasts through P38/ERK1/2 activation [63]. Furthermore, JNK and p38 can be phosphorylated in LPS-induced inflammatory responses in L6 myoblasts [44]. In line with the above findings, our results showed that LPS triggered the phosphorylations of p38, JNK, and ERK with distinct patterns. However, H_2_S decreased the phosphorylations of these protein kinases. These results together suggest that H_2_S can decrease ROS-induced apoptosis through the MAPK signaling pathway in LPS-treated L6 cells.

It has been shown that LPS could increase expressions of inflammatory cytokines in L6 cells, such as TNF-*α*, MCP-1, IL-6, and IL-10 [43–45]. In the present study, LPS treatment increased the expression levels of MCP-1, TNF-*α*, IL-1*β*, IL-6, IL-10, and IL-18. H_2_S treatment decreased the levels of these inflammatory cytokines, indicating that H_2_S is capable of alleviating inflammation in LPS-treated L6 cells. TLR4, a main receptor of LPS, plays a key role in the initiation and acceleration of inflammatory responses induced by LPS [64, 65]. Activation of TLR4 by LPS can induce the activation of the NF-*κ*B signaling pathway to regulate the release of proinflammatory cytokines [46, 47]. Our results showed that LPS increased the expressions of TLR4, p-TAK1, p-IKK*α*/*β*, p-I*κ*B*α*, p50, and p-p65. However, these effects were reversed by treatment with H_2_S, suggesting that H_2_S is able to reduce LPS-induced inflammation by suppressing the TLR4/NF-*κ*B signaling pathway in L6 cells.

Recent studies have demonstrated that administration of LPS can induce diaphragmatic contractile dysfunction in several animal models [24, 27, 29]. Our results indicated that LPS treatment reduced the force-generating capacity, maximum tetanic tension, peak twitch tension, fatigue index, and the maximal rates of contraction and relaxation of the diaphragm of septic rats. Administration of H_2_S improved the impaired diaphragmatic function induced by LPS in septic rats. In addition, a recent study has shown that LPS treatment decreases the CSA of embryonic MHC fibers in the preterm diaphragm of pregnant ewes [66]. Our results showed that LPS decreased the CSA of both MHC_slow_ and MHC_fast_, while H_2_S prevented LPS-induced loss of CSA of MHC_slow_ and MHC_fast_ in the diaphragm of septic rats. Furthermore, H_2_S decreased levels of inflammation and apoptosis induced by LPS in the diaphragm of septic rats. These data together indicate that H_2_S is capable of improving diaphragmatic function and structure through reduction of inflammation and apoptosis in the diaphragm of septic rats.

In conclusion, our results demonstrate that H_2_S is able to ameliorate LPS-induced diaphragm dysfunction in rats by reducing apoptosis and inflammation through ROS/MAPK and TLR4/NF-*κ*B signaling pathways. Novel slow-releasing H_2_S donors can be designed and applied for the treatment of diaphragm dysfunction.

## Figures and Tables

**Table 1:** Primers used in this study.

Genes	Sense primers (5′–3′)	Antisense primers (5′–3′)
TNF-*α*	GAAAGCATGATCCGAGATGTGGAA	CAGTAGACAGAAGAGCGTGGTGGC
MCP-1	CTATGCAGGTCTCTGTCACGCTTC	CAGCCGACTCATTGGGATCA
IL-1*β*	TGCAGGCTTCGAGATGAA	AGGCCACAGGGATTTTGTC
IL-6	ACAGCCACTGCCTTCCCTAC	GAATTGCCATTGCACAACTCTT
IL-10	CCTCTGGATACAGCTGCGAC	CAGTAGATGCCGGGTGGTTC
IL-18	GACCGAACAGCCAACGAATC	GATAGGGTCACAGCCAGTCC
GAPDH	CGTGTTCCTACCCCCAATGTA	CACAGGAGACAACCTGGTCC

## Data Availability

The raw data used to support the findings of this study are available from the corresponding author upon request.
